# Activation of endogenous tolerance to bleaching stress by high salinity in cloned endosymbiotic dinoflagellates from corals

**DOI:** 10.1186/s40529-025-00451-5

**Published:** 2025-01-15

**Authors:** Ching-Nen Nathan Chen, Tze Ching Yong, Jih-Terng Wang

**Affiliations:** Department of Oceanography, National Sun Yat-Sen University, Kaohsiung, 804 Taiwan

**Keywords:** Symbiodiniaceae, Reactive oxygen species, Symbiotic dinoflagellates, Coral bleaching

## Abstract

**Background:**

Large-scale coral bleaching events have become increasingly frequent in recent years. This process occurs when corals are exposed to high temperatures and intense light stress, leading to an overproduction of reactive oxygen species (ROS) by their endosymbiotic dinoflagellates. The ROS buildup prompts corals to expel these symbiotic microalgae, resulting in the corals’ discoloration. Reducing ROS production and enhancing detoxification processes in these microalgae are crucial to prevent the collapse of coral reef ecosystems. However, research into the cell physiology and genetics of coral symbiotic dinoflagellates has been hindered by challenges associated with cloning these microalgae.

**Results:**

A procedure for cloning coral symbiotic dinoflagellates was developed in this study. Several species of coral symbionts were successfully cloned, with two of them further characterized. Experiments with the two species isolated from *Turbinaria* sp. showed that damage from light intensity at 340 μmol photons/m^2^/s was more severe than from high temperature at 36 °C. Additionally, preincubation in high salinity conditions activated their endogenous tolerance to bleaching stress. Pretreatment at 50 ppt salinity reduced the percentage of cells stained for ROS by 59% and 64% in the two species under bleaching stress compared to those incubated at 30 ppt. Furthermore, their Fv’/Fm’ during the recovery period showed a significant improvement compared to the controls.

**Conclusions:**

These findings suggest that intense light plays a more important role than high temperatures in coral bleaching by enhancing ROS generation in the symbiotic dinoflagellates. The findings also suggest the genomes of coral symbiotic dinoflagellates have undergone evolutionary processes to develop mechanisms, regulated by gene expression, to mitigate damages caused by high temperature and high light stress. Understanding this gene expression regulation could contribute to strengthening corals’ resilience against the impact of global climate change.

**Supplementary Information:**

The online version contains supplementary material available at 10.1186/s40529-025-00451-5.

## Background

Colorful coral reefs are among the most productive and biologically diverse ecosystems, home to a complex web of marine species. The foundation of this productivity and diversity is an endosymbiotic relationship between corals and a taxon of dinoflagellates, a type of microalgae that can form either specific or nonspecific associations with their coral hosts (Caruso et al. 2021). These dinoflagellates carry out photosynthesis, producing organic carbon that sustains the metabolic demands of the corals. In return, the corals provide a safe, nutrient-rich environment, supplying vital compounds like organic nitrogen and phosphate that support the algae’s biological functions (Muller-Parker et al. 2015). This interdependence is crucial for reef health, as the energy produced by the dinoflagellates enables corals to grow and deposit calcium carbonate, building the structural groundwork of the reefs that shelter diverse organisms.

Coral bleaching is a stress response in which corals expel these symbiotic dinoflagellates, leading to a noticeable whitening of coral tissues. Large-scale bleaching events typically occur when surface seawater temperature remain elevated for prolonged periods, often aggravated by intense sunlight (Lesser 2011). Without their symbiotic algae, corals are deprived of a major source of organic carbon, leaving them vulnerable to disease and nutrient scarcity, as their capacity to capture plankton from the water alone is insufficient. If this symbiosis is not restored in time, corals may weaken and die, resulting in habitat degradation and potential collapse of reef ecosystems. Such a collapse has far-reaching implications, as coral reefs support numerous marine species and sustain local economies through tourism and fisheries. Continued global warming and increased bleaching frequency threaten the stability of these ecosystems profoundly, underscoring the urgent need for conservation strategies to protect these vital habitats (Brandl et al. 2019).

The overproduction of reactive oxygen species (ROS) during photosynthesis in endosymbiotic dinoflagellates under high temperatures and high light stress can lead to expulsion or death of these coral symbiotic microalgae, disrupting coral health and causing coral bleaching (Levin et al. 2016; McGinty et al. 2012; Wietheger et al. 2018). Photosystem II (PSII) and Photosystem I (PSI), key protein complexes in the photosynthetic pathway, are major sources of ROS. In PSII, water molecules are split to release electrons, protons, and oxygen. These electrons are subsequently transferred to PSI via carrier molecules in the electron transport chain, where they receive another energy boost, enabling further reactions. However, under stress conditions such as high light and elevated temperatures, excess electrons can escape regulation to interact with oxygen and form superoxide radicals (^.^O_2_) (Foyer and Shigeoka 2011; Oakley and Davy 2018). Superoxide radicals can be further converted into hydrogen peroxide (H_2_O_2_) via the enzyme superoxide dismutase (SOD), and in turn, hydrogen peroxide can react with superoxide to produce hydroxyl radicals (^.^OH^−^), which are among the most reactive and damaging molecules in biological systems (Chen and Pan 1996; Nishiyama et al. 2006). These ROS are highly reactive and can cause cellular damage through various mechanisms such as lipid peroxidation of polyunsaturated fatty acids, DNA fragmentation, and protein denaturation. High light intensity poses additional risk by damaging the D1 protein in PSII irreversibly; this damage is believed to result from singlet oxygen production in PSII under excessive irradiance, leading to impaired photosynthetic efficiency (Hideg et al. 2002; Nishiyama et al. 2006). As a result, the buildup of ROS creates a toxic environment for both the dinoflagellate symbionts and coral host cells, which ultimately contributes to the breakdown of the symbiotic relationship and triggers coral bleaching.

Effective strategies to reduce ROS production or enhance ROS detoxification efficiency in coral symbiotic dinoflagellates are essential for preventing coral bleaching and conserving coral reef ecosystems. For this purpose, stable cell lines of cloned endosymbiotic dinoflagellates are required. However, successful cloning of coral symbionts has rarely been reported. The scarcity of high-quality cell lines has significantly hindered studies on the cell physiology and genetic transformation of the symbionts, as well as investigations into coral-symbiont interactions. To accelerate these studies, we developed a cloning strategy through trial and error. Challenges in cloning and culturing symbiotic dinoflagellates included physiological shock from host cell disruption (Goiran et al. 1997; Wang et al. 2011), susceptibility to bacterial or fungal infections, and predation by protozoa such as ciliates. These issues were addressed in this study.

Several strains of coral symbionts were successfully cloned and proliferated using our newly developed method, and two of these isolates were further characterized. In the following cell physiology study we found that high salinity remarkably increased the tolerance of these cells to environmental stressors such as elevated temperature and intense light. This finding is crucial, as understanding the molecular mechanisms—such as the regulatory genes underlying this stress tolerance—could allow us to enhance the resilience of coral symbionts. Through approaches such as mutant selection, genetic transformation, or gene editing, we may strengthen the stress tolerance of these symbionts, helping to prevent coral bleaching in the warming oceans.

## Materials and methods

### Cloning and proliferation of symbiotic dinoflagellates from corals

A piece of stony coral *Turbinaria* sp., a gift from Dr. P. J. Meng of the National Museum of Marine Biology and Aquarium of Taiwan, was rinsed with filtered artificial seawater (ASW) a few times to clean its surface contamination. The coral cells were then smashed by spreading high-pressure ASW using a painter’s airbrush powered by an air compressor set at 90 to 110 psi (appendix Fig. 6). The coral debris and the released symbiotic microalgae, collected in a plastic bag when smashing, were diluted to 25 mL and filtered through a layer of Kimwipes paper (Kimberly-Clark Global Sales Inc., USA) supported by a layer of 25 μm mesh nylon filter in a funnel (appendix Fig. 7). After filtration the cells were settled down by using low speed centrifugation (100 × *g* for 10 min) in the 50-mL tube and the supernatant was discarded immediately by suction to remove micro-debris and macromolecules from the smashed tissues. This cleaning step was repeated once. These cells were then resuspended in filtered ASW (note: recently we found 40 ppt ASW worked better than 30 ppt ASW) containing 36 mg IMK/L (Daigo’s IMK Medium, Fujifilm Waco Chemicals, Japan) and 200 μM glycine (referred to as ASW-IMK-glycine medium hereafter). Density of these suspended cells was adjusted to 5 to 10 cells per major square examined by using a hemocytometer, which houses 0.1 μL of the suspension in the square. If over-diluted, the medium can be removed using the procedure of the cleaning step. The cell density at this step is about 10,000–20,000 cells per 200 μL. This suspension was further serially diluted to no more than one cell per 200 μL medium. Two hundred μL of the cell suspension was dispensed to each well of medical grade 96-well plates. Another 100 μL fresh ASW-IMK-glycine medium was added to each well to make 300 μL per well. These plates were kept in the dark at 25 °C for the first week, followed by illumination of 20 μmol photon/m^2^/s with day/night cycle of 14/10 h until cell growth could be observed by naked eyes in some wells, which would require about 3 months. The clones with significant cell density in the 96-well plates, verified using an inverted microscope, were transferred to new 24-well cell culture plates, and the culture medium and the light intensity were changed to 2 mL ASW-IMK medium and 50 μmol photon/m^2^/s, respectively as described below in the cell cultivation conditions section. The clones were further transferred to 125 mL flasks containing 20 mL ASW-IMK medium when their cell densities were significant. The ASW-IMK medium in the flasks was renewed every month.

### Symbiotic dinoflagellate cultivation conditions and stress treatments

For cell culture maintenance, coral symbiotic dinoflagellates were grown in artificial seawater (ASW) prepared by dissolving 30 g sea salt mix (Instant Ocean, Aquarium Systems, France) in 1 L water. After overnight precipitation, the ASW was filtered through a 0.22 μm pore size filtration cup (Nalgene, Thermo Scientific, USA) facilitated by a vacuum. The IMK nutrient mix at 250 mg/L was added to filtered ASW before use (referred to as ASW-IMK medium hereafter). The cells were maintained in a growth chamber conditioned at 50 μmol photon/m^2^/s white light from 9 am to 11 pm a day (day/night = 14/10 h) at 25 °C. To prepare artificial seawater with higher levels of salinity, the sea salt mix was weighed to the desired amount and dissolved in water. To prepare cells for the stress treatments, cells grown to early stationary phase were harvested and incubated in 30, 40, or 50 ppt salinities ASW without addition of nutrients for three days at 25 °C and 50 μmol photon/m^2^/s followed by the treatments. In the high temperature treatments, the temperatures during the daytime and the nighttime were 36 and 28 °C, respectively. Dark treatment was carried out by wrapping the plates in aluminum foils and placed together with the controls in the same growth chamber. The double stress treatment in this study referred to high temperature and high light conditions in the growth chamber settings.

### DNA extraction, PCR and sequencing

Aliquots of symbiotic dinoflagellate clones were transferred to separate microtubes. Their cell densities were estimated using a hemocytometer and the total cell number of each aliquot calculated. After replacing the medium with freshwater, immediately the cells were boiled for 5 min to inactivate enzymes in the cells. The dead cells were smashed using a mini-beadbeater, centrifuged at 16,000 × *g* for 10 min, and the supernatant of each sample was transferred to a new microtube. For a PCR reaction, an amount of the supernatant containing the DNA extracted from about 300 cells was used. The PCR was carried out using the conventional protocol. The primers’ sequences for amplification of the D1/D2 region of the symbiotic microalgae 28S rDNA were 28SzooxD1/D2F: 5’-CCT CAG TAA TGG CGA ATG AAC A-3’ and 28SzooxD1/D2R: 5’-CCT TGG TCC GTG TTT CAG GA-3’ (Lien et al. 2007; Loh et al. 2002). The sequencing was carried out using the dideoxy method. The partial 28S rDNA sequences of the two strains were used to BLAST against the GenBank. Similar sequences were selected, aligned, and trimmed to construct the phylogenetic tree.

### Phylogenetic analysis

The evolutionary history was inferred using the Neighbor-Joining method (Saitou and Nei 1987), and the optimal tree is shown. The percentage of replicate trees in which the associated taxa clustered together in the bootstrap test (1000 replicates) are shown next to the branches. The tree is drawn to scale, with branch lengths in the same units as those of the evolutionary distances used to infer the phylogenetic tree. The evolutionary distances were computed using the Kimura 2-parameter method (Kimura 1980) and are in the units of the number of base substitutions per site. The rate variation among sites was modeled with a gamma distribution (shape parameter = 1). This analysis involved 13 nucleotide sequences. All ambiguous positions were removed for each sequence pair (pairwise deletion option). There was a total of 601 positions in the final dataset. Evolutionary analyses were conducted in MEGA X (Kumar et al. 2018).

### Cell count, scanning electron microscopy (SEM) and measurement of photosystem II efficiency (Fv’/Fm’)

The procedure for carrying out SEM was described in Chiu et al. (Chiu et al. 2020). For the measurement of photosystem II efficiency (quantum yield, Fv’/Fm’), the cells were cultivated in 1 L flasks containing 150 mL of the ASW-IMK medium until mid-log phase in conditions described above. A portion of the culture was harvested and its medium was renewed followed by cell density measurement using a particle analyzer (Multisizer III, Beckman Coulter, USA). Aliquots of the prepared cells were transferred to medical grade 24-well plates, each well received 500 thousand (0.5 × 10^6^) cells. The volume of the medium in each well was brought to 2 mL using the ASW-IMK medium. These cells were incubated at 25 °C under 50 μmol photon/m^2^/s light intensity for three days before the stress treatments. Light-adapted photosystem II (PSII) efficiency (Fv’/Fm’) was measured by a portable fluorometer (AquaPen AP-C 100, Photon Systems Instruments, Czech Republic). The procedure was described in Pan et al. (2011).

### Detection of reactive oxygen species (ROS) using fluorescent dye

The fluorescent dye 2’, 7’- dichlorofluorescin diacetate (H_2_DCFDA) was used to detect ROS in the cells (Oparka et al. 2016). The procedure was described in Wang et al. (2011). Briefly, H_2_DCFDA was dissolved in dimethylsulfoxide (DMSO) to prepare 10 mM solution. Cells in the early stationary phase were used for the staining and five μL of the solution was added to 1 mL of cell suspension. This mix was kept in the dark for 30 min followed by fluorescent microscopy observation and imaging. At least 500 cells in each treatment were imaged and counted to obtain the ratio of the stained cells.

## Results

### An effective method for the cloning and proliferation of coral symbiotic dinoflagellates and initial characterization

**Fig. 1:**
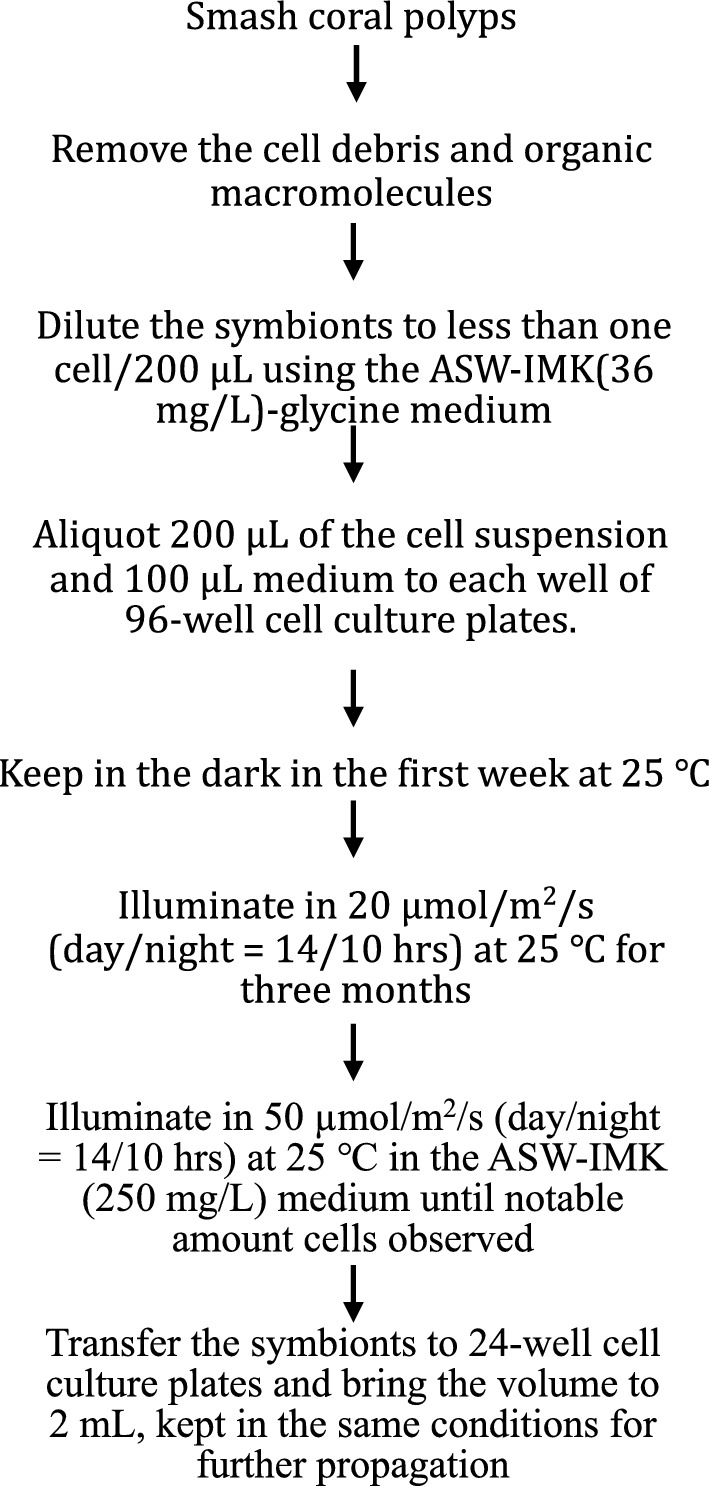
Schematic diagram of the coral symbiotic dinoflagellate cloning procedure

Polyps present on an approximately 2 cm^2^ piece of the flat stony coral *Turbinaria* sp. were smashed by spraying artificial seawater (ASW) at high pressure. The symbiotic dinoflagellates released from the coral cells were collected as described in Materials and Methods and the schematic diagram in Fig. 1. Approximately 40 mL of the diluted symbiont suspension was aliquoted to the wells of 96-well cell culture plates. In the first successful cloning trial, coral symbiont growth was observed in 32 out of 192 wells (two plates). However, the proportion of wells with symbiont growth was lower in the second trial, only 18 out of 192, performed using the same coral species. In the two follow-up trials using this newly developed method, we successfully cloned symbionts from *Acropora* sp. and *Stylocoeniella* sp.; the successful rates were 40/192 and 36/192, respectively.

**Fig. 2:**
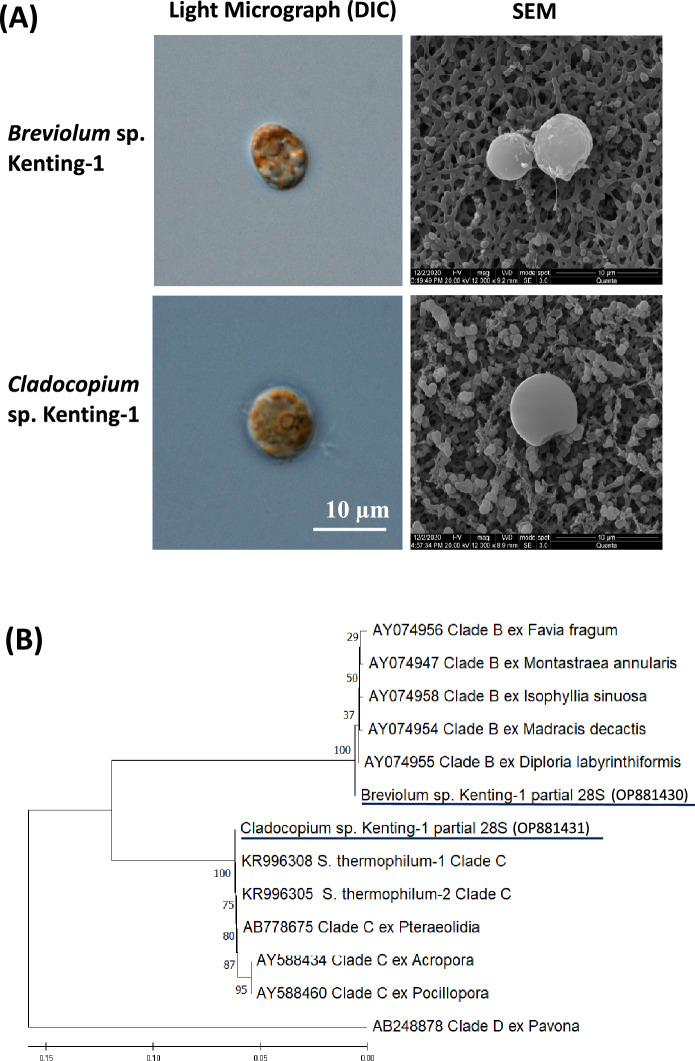
**A** Micrographs of *Breviolum* sp. Kenting-1 and *Cladocopium* sp. Kenting-1 imaged using Differential Interference Contrast (DIC) light microscopy and Scanning Electron Microscopy (SEM). **B** Phylogenetic analysis of *Breviolum* sp. Kenting-1, *Cladocopium* sp. Kenting-1 and their closely related species based on their 28S rDNA sequences in the D1/D2 region that contained 601 nucleotides. The GenBank accession numbers of these sequences are shown in the tree

In the subsequent proliferation of isolates from *Turbinaria* sp., we found that the clones exhibited two swimming patterns: S-shape and spiral. Genomic DNA was isolated from these clones, and their 28S rDNA in the D1/D2 region was amplified and sequenced (Lien et al. 2007; Loh et al. 2002). Their DNA sequences showed that these clones could be divided into two species, in line with their swimming patterns. Micrographs of the two new symbiont species were taken using Differential Interference Contrast (DIC) light microscopy and Scanning Electron Microscopy (SEM) (Fig. 2A). Similar to other coral symbionts, the cells of these two symbiont species contained prominent pyrenoids and many chloroplasts. A comparison of our sequencing results with the relevant sequences in GenBank revealed that these clones belonged to two genera of the Symbiodiniaceae family: *Breviolum* and *Cladocopium*. Phylogenetic analysis (Fig. 2B) indicated that the D1/D2 region in the 28S rDNA sequence differed between *Breviolum* and *Cladocopium*. Videos showing the swimming patterns of the isolates *Breviolum* sp. Kenting-1 and *Cladocopium* sp. Kenting-1 have been uploaded on YouTube (https://www.youtube.com/shorts/9eUj4CxPFRY and https://www.youtube.com/shorts/wFQE45pwvq8, respectively), and their partial 28S rDNA sequences have been deposited in GenBank under the accession numbers OP881430 and OP881431, respectively.

### High salinity induced protective mechanisms in coral symbiotic dinoflagellates against high temperature and high light intensity

**Fig. 3:**
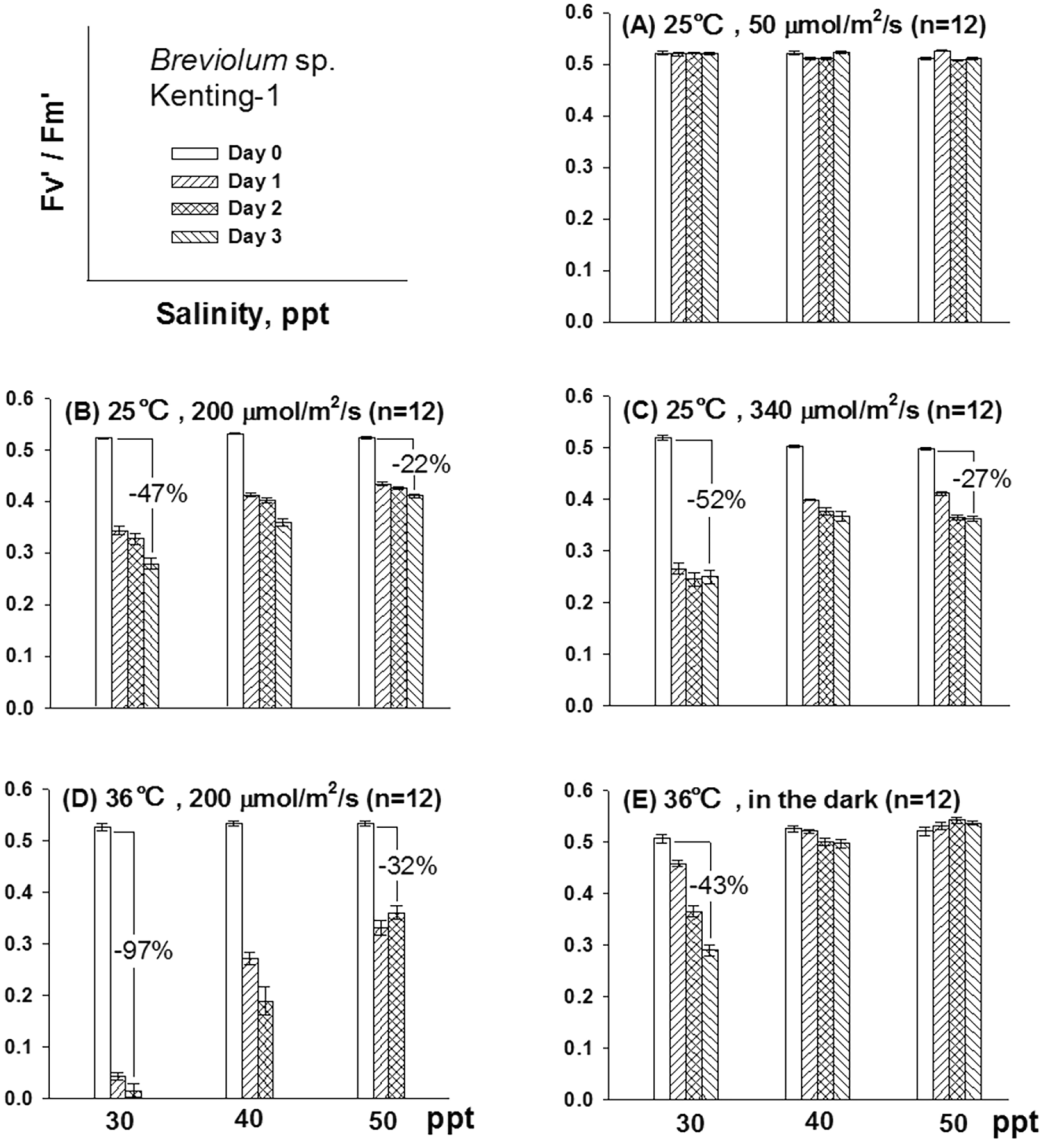
Changes in the light-adapted photosystem II (PSII) efficiency (Fv’/Fm’) of *Breviolum* sp. Kenting-1 cells treated under different conditions of temperature, light intensity, and salinity for 3 days (n = 12, mean ± SE). Day/night = 14/10 h. **A**–**C**, temperature (day/night) at 25 °C; **D** and **E**, 36 °C (day) and 28 °C (night). Before the stress treatments, these cells were incubated at 30, 40, or 50 ppt salinity at 25 °C in 50 μmol photon/m^2^/s for 3 days

During a mutant selection work, we observed that incubating the cells in a highly saline medium protected the symbionts against the stress of high temperature and high light intensity. This observation prompted a further investigation. Because PSII efficiency is sensitive to environmental stress (Nishiyama et al. 2006), the light-adapted PSII efficiency (Fv′/Fm′) was used as an indicator to measure the protective effect of high salinity (Pan et al. 2011). *Breviolum* sp. Kenting-1 incubated at 30, 40, and 50 ppt salinity under 50 μmol photon/m^2^/s at 25 °C maintained the same levels of Fv′/Fm′ for 3 days (Fig. 3A). When light intensity was increased to 200 μmol photon/m^2^/s, the symbionts incubated at 40 and 50 ppt salinity exhibited higher PSII efficiency than those incubated at 30 ppt (Fig. 3B). In this stress experiment, we noted a 47% reduction in Fv′/Fm′ in the symbionts incubated at 30 ppt salinity from Day 0 to 3 compared with a 22% reduction in those incubated at 50 ppt salinity during the same period.

In another stress experiment, when light intensity was increased to 340 μmol photon/m^2^/s, more severe reduction in Fv′/Fm′ was noted in all groups (Fig. 3C). We observed a 52% reduction in Fv′/Fm′ in the cells incubated at 30 ppt salinity from Day 0 to 3 compared with a 27% reduction in those incubated at 50 ppt salinity during the same period.

To investigate the protective effect of high salinity on the symbionts, we incubated these cells under double stress conditions (200 μmol photon/m^2^/s at 36 °C daytime and 28 °C nighttime). Large reductions in Fv′/Fm′ were observed in the cells incubated at 30 ppt salinity on Day 1 and 2, being reduced to a non-detectable level on Day 3. The symbionts incubated at 40 or 50 ppt salinity exhibited moderate reductions in Fv′/Fm′ on Day 1 and 2, being reduced to a non-detectable level on Day 3 as well (Fig. 3D). A 97% reduction in Fv′/Fm′ was observed in the cells incubated at 30 ppt salinity from Day 0 to 2; only 32% reduction was noted in the cells incubated at 50 ppt salinity during the same period. To differentiate the stress effect of light from temperature, the cells were treated at 36 °C in the dark. Minor reductions in Fv′/Fm′ were observed in the cells incubated at 40 ppt salinity from Day 0 to Day 3 (Fig. 3E), and no reduction noted in the cells incubated at 50 ppt during the same period. By contrast, a 43% reduction in Fv′/Fm′ was observed in the cells incubated at 30 ppt salinity during the same period. Similar patterns of stress responses were observed in *Cladocopium* sp. Kenting-1 (Supplementary Fig. 1).

### Coral symbiotic dinoflagellates incubated in a highly saline medium obtained better protection and exhibited better recovery from cellular damage

**Fig. 4:**
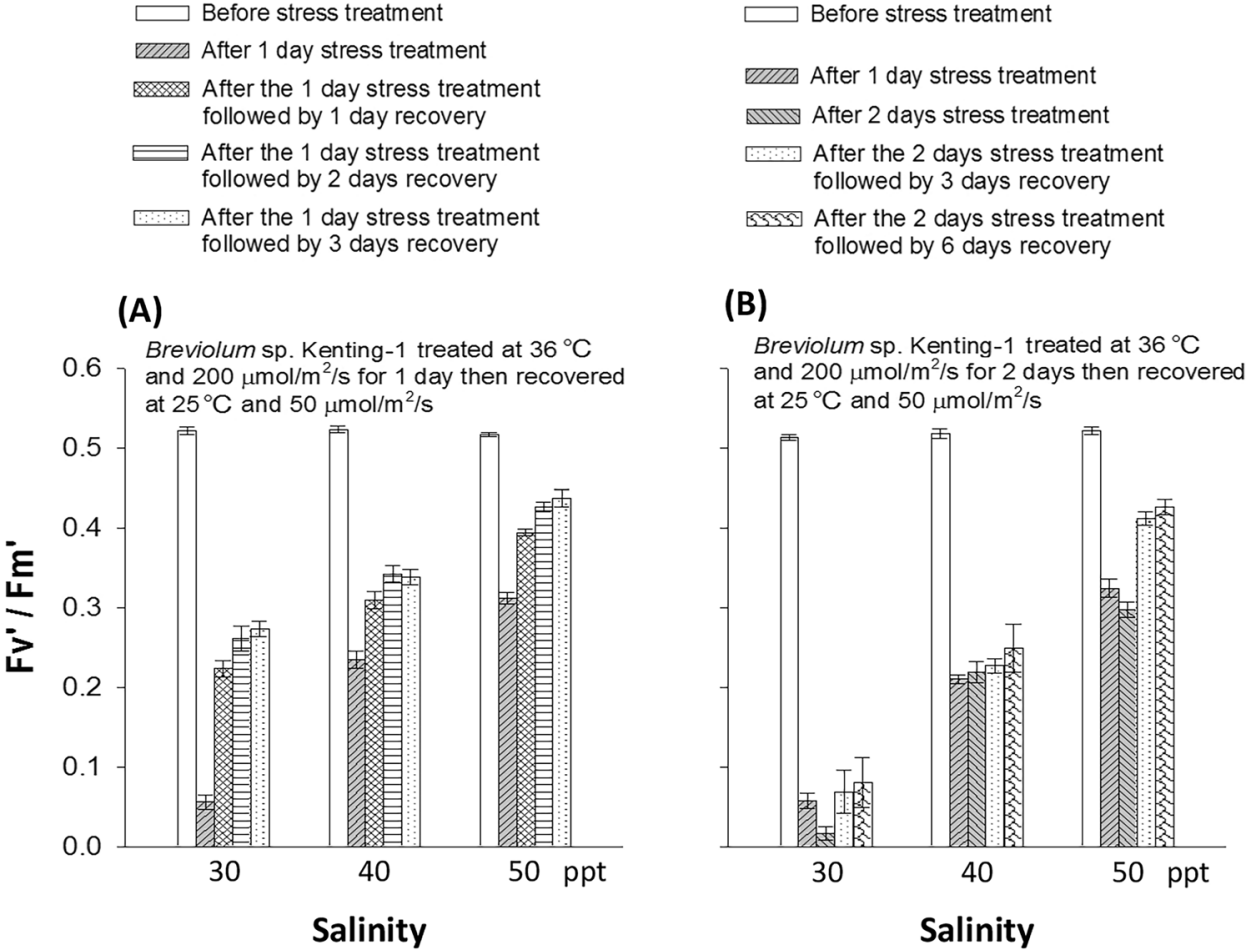
Recovery of the light-adapted photosystem II (PSII) efficiency (Fv’/Fm’) of *Breviolum* sp. Kenting-1 after the stress treatment (36 °C with 200 μmol photon/m^2^/s at daytime and 28 °C at night, day/night = 14/10 h) for one day (**A**) or two days (**B**) at different levels of salinity (n = 12, mean ± SE)

Treating the symbionts under the double stress conditions (36 °C plus 200 μmol photon/m^2^/s) considerably reduced the PSII efficiency of the two species (Fig. 3 and Supplementary Fig. 1). However, the low Fv′/Fm′ levels may not imply death of the symbionts. One possible explanation for this finding is that some photosynthetic pigments and proteins were degraded as a protection mechanism under the double stress; however, the cells were still alive. In this scenario, the stressed cells would recover after the stress is relieved. To verify this possibility, *Breviolum* sp. Kenting-1 cells incubated at 30, 40, and 50 ppt salinity were treated under the double stress conditions for 1 day (Fig. 4A; the conditions and day/night cycle are described in Materials and Methods) or 2 days (Fig. 4B). These experiments were followed by recovery periods, during which they were incubated under normal conditions (25 °C and 50 μmol photon/m^2^/s). As shown in Fig. 4A, after exposing the cells to the double stress conditions for 1 day, a significant reduction was observed in the Fv′/Fm′ level of the symbionts incubated at 30 ppt salinity, and this reduction was greater than that observed for cells incubated at 40 and 50 ppt salinity as expected. All three groups of cells recovered substantially after incubation under normal conditions for 3 days. As shown in Fig. 4B, after incubation under the double stress conditions for 2 days, the symbionts incubated at 30 ppt salinity had difficulty in regaining their PSII activity even after 6 days of recovery. For the symbionts incubated at 40 and 50 ppt salinity, the Fv′/Fm′ level did not decrease to the level similar to that observed in cells incubated at 30 ppt salinity. Moreover, the symbionts incubated at 50 ppt salinity recovered to 80% of the pre-stress level after incubation under normal conditions for 6 days. However, the symbionts incubated at 40 ppt salinity did not exhibit notable recovery.

The recovery patterns of *Cladocopium* sp. Kenting-1 exposed to the double stress conditions for 1 day (Supplementary Fig. 2A) were similar to those of *Breviolum* sp. Kenting-1. However, differences were observed in the recovery after their exposure to the double stress conditions for 2 days (Supplementary Fig. 2B). The Fv′/Fm′ level of the symbionts incubated at 30 ppt salinity was not detected after the 2-day-long exposure to the double stress conditions; no sign of recovery was observed over the following 6 days of incubation under normal conditions. These cells were found to be dead when examined under a fluorescent microscope. The Fv′/Fm′ level of the symbionts incubated at 40 ppt salinity was not detected after the 2-day-long exposure to the double stress conditions either. However, approximately 10–15% of the Fv′/Fm′ level was regained after 6 days of recovery. In the symbionts incubated at 50 ppt salinity, approximately 45% of the Fv′/Fm′ level was retained after the 1-day-long exposure to the double stress conditions. However, the Fv′/Fm′ level was not detected after the 2-day-long exposure to the double stress conditions. Approximately 60% of the Fv′/Fm′ level was regained after 6 days of recovery. Taken together, these results indicated that high salinity (50 ppt) not only protected coral symbionts from the double stress of high temperature and high light intensity but also facilitated their recovery when the stress was relieved. Whether the mechanisms of protection and recovery were controlled by the same set of subcellular activities require further investigation.

### Coral symbiotic dinoflagellates incubated at 50 ppt salinity produced lower levels of ROS than those incubated at 30 ppt salinity

Prolonged elevated surface seawater temperatures can induce stress in coral symbionts, leading to increased ROS production and ultimately to coral bleaching (Baird et al. 2018; Oakley and Davy 2018). However, coral bleaching is often triggered by the combined effects of high temperature and intense light exposure. Further, as shown in Fig. 3C, E, the damage caused by high light stress at 340 μmol photons/m^2^/s was more severe than that caused by high temperature stress at 36 °C in the dark. To investigate the involvement of ROS in this damage and whether high salinity activates endogenous protective mechanisms, cells of the two symbiotic dinoflagellate species were incubated in either 30 or 50 ppt salinity for 3 days. This was followed by exposure to 36 °C with or without 200 μmol photons/m^2^/s light stress (ref. Figure 3D), after which cells were stained to assess ROS levels.

**Fig. 5:**
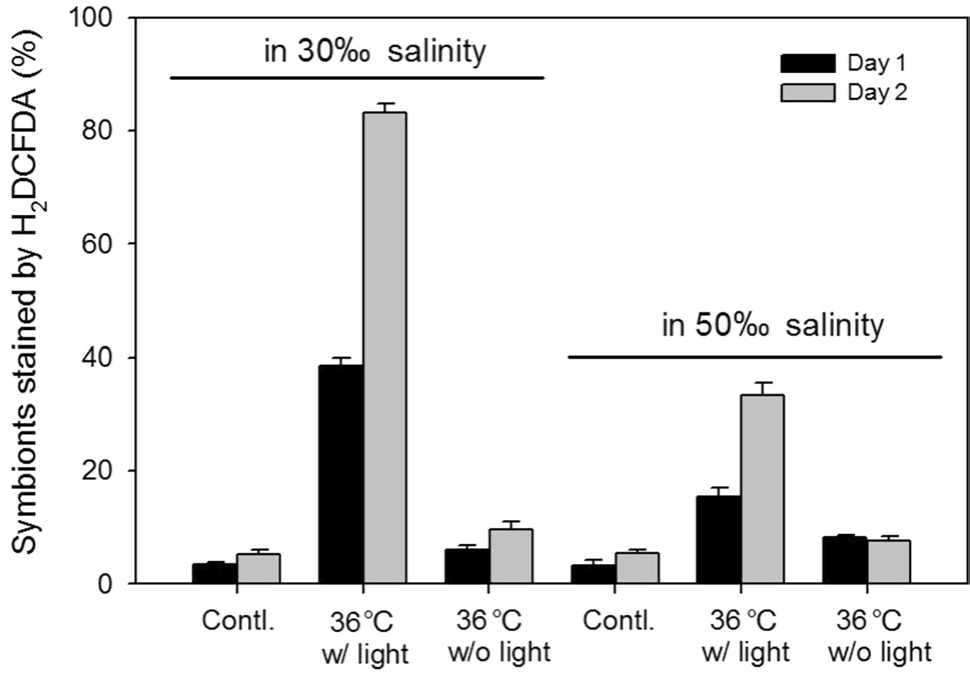
Proportions of *Breviolum* sp. Kenting-1 cells stained by the fluorescent dye H_2_DCFDA in different conditions (n = 3, mean ± SE). Cells in the experimental groups were treated at 30 or 50 ppt salinity coupled with (w/) 200 μmol photon/m^2^/s light intensity or without (w/o) light at 36 °C. The control groups (Contl.) were treated at 25 °C coupled with 50 μmol photon/m^2^/s light intensity at 30 or 50 ppt salinity

To visualize ROS, the cells were stained using the fluorescent dye 2′,7′-dichlorofluorescin diacetate (H_2_DCFDA)(Wang et al. 2011). H_2_DCFDA reacts with ROS and undergoes oxidation. When excited by blue light, the oxidized form of H_2_DCFDA emits strong green light (Oparka et al. 2016), which is visible under a fluorescence microscope. To quantify the proportion of cells emitting green light, more than 500 cells were imaged and counted for each treatment. After exposure to the double stress conditions, a significantly higher proportion of cells of both symbiont species incubated at 30 ppt salinity produced detectable levels of ROS than those incubated at 50 ppt salinity (Fig. 5 and Supplementary Fig. 3). Compared with those incubated at 30 ppt salinity for 2 days, the reduction of the stained cells was 59% in *Breviolum* sp. Kenting-1 and 64% in *Cladocopium* sp. Kenting-1 incubated at 50 ppt salinity, respectively. However, exposure to the single stress of high temperature (36 °C) did not lead to a marked difference in H_2_DCFDA staining results between the cells incubated at 30 ppt salinity and those incubated at 50 ppt salinity in each species, and the proportions of the stained cells were significantly lower than those groups treated under the double stress. These findings, together with the results in Fig. 3E and Supplementary Fig. 1E, support the notion that high light intensity plays a more damaging role than high temperatures in coral bleaching by enhancing ROS generation in coral symbionts.

## Discussion

Frequent high temperatures, often coupled with intense light, in surface seawater pose significant threats to coral reef ecosystems worldwide. As these ecosystems support approximately 25% of all marine species, large-scale coral bleaching has the potential to drive many of these species toward extinction, underscoring the importance of preventing coral bleaching to ensure sustainable reef protection and development. A primary cause of bleaching under high temperature and light conditions is the overproduction of reactive oxygen species (ROS), such as superoxide, hydrogen peroxide, and hydroxyl radicals, within the photosynthetic processes of symbiotic dinoflagellates in coral hosts. Reducing ROS production and enhancing ROS scavenging in symbionts are therefore essential strategies for preventing coral bleaching. Among the various approaches to cope with ROS, an effective method involves mutagenesis combined with artificial selection to develop stress-tolerant coral symbionts. The coral symbiont cloning method presented in this study offers a practical approach for coral research groups across different regions to participate in mutagenesis-artificial selection studies. The selected strains with enhanced thermal and high light tolerance could potentially be reintroduced to coral hosts, expediting natural selection, which is typically a very slow process. Additionally, we found that high salinity can increase thermal and light tolerance in coral symbionts. This finding suggests that coral symbiotic dinoflagellates may have evolved genetic mechanisms that confer tolerance to high temperature and high light stress. Understanding these tolerance-enhancing mechanisms could provide valuable insights for preventing coral bleaching. This intrinsic stress tolerance is reminiscent of that observed in some resilient corals in highly saline, warm waters, such as those in the Red Sea. Future studies should investigate whether the resilience of Red Sea corals is a result of high salinity.

Key technical aspects of the symbiotic dinoflagellate cloning procedure are detailed below, as this is a novel protocol for coral symbiont cloning and no alternative reference available. Disrupting coral tissues to release their symbionts produced substantial amounts of organic debris and macromolecules. If not thoroughly removed, these organic materials could serve as nutrients for bacterial and fungal growth, potentially harming the symbionts. To remove larger organic matter, filtration was performed using Kimwipes paper on top of a 25 μm mesh nylon screen. To remove micro-debris and macromolecules, since their precipitation rates are slower than that of the symbionts, the supernatant containing these fine particles was removed by suction after most of the symbionts had settled at the bottom of the collection tube. Subsequently, the symbionts were diluted to reduce cell density to fewer than one eukaryotic cell per 200 µL, further decreasing the levels of organic matter in the suspension. This dilution also eliminated protozoan grazing, as no more than one cell from the suspension was transferred to each well. Recognizing that the symbionts were freshly released from their host cells and faced a major environmental change, we minimized potential stress by performing the cloning under low light and placing the symbionts in 96-well plates in the dark for 1 week to allow gradual adaptation to their new environment. During this dark incubation period, glycine was added to the medium as a source of nitrogen and carbon. This incubation in glycine-containing medium under dark conditions was essential for successful cell cloning. After 1 week in the dark, the cells were exposed to 20 µmol photons/m^2^/s light intensity (14/10-h day/night cycle) at 25 °C. It took 2–3 months for a single cell to proliferate into a visible population. Prior to the formation of visible colonies, the cells were observed under an inverted microscope, with minimal light exposure and short observation times to prevent stress. Methods for cloning coral symbionts have been presented in previous studies (Beltran et al. 2021; LaJeunesse and Parkinson 2012). In comparison, the procedure developed in this study is both simpler and more effective. This method has been successfully used to clone symbiotic dinoflagellates from *Turbinaria* sp., *Acropora* sp., *Stylocoeniella* sp., and *Leptoria* sp. in two independent laboratories.

The observation that elevated salinity enhances the tolerance of two coral symbiont species to high temperature and intense light is consistent with the concept of cross-tolerance observed in plants. In this phenomenon, stress tolerance induced by one environmental factor confers protection against other stressors. For instance, drought-induced tolerance has been shown to protect plants from low-temperature stress, and vice versa (Pastori and Foyer 2002; Zhu 2000). Although the mechanisms underlying salinity-induced tolerance in coral symbionts remain poorly understood, they are likely linked to the upregulation of protective proteins. These may include reactive oxygen species (ROS)-scavenging enzymes, small antioxidant biosynthesis enzymes, heat shock proteins, molecular chaperones, and fatty acid desaturases. The identification of transcription factors that regulate the expression of these proteins is crucial, as advancements in gene editing technologies could be applied to coral symbionts in the future. Through the controlled manipulation of key transcription factors, it may be feasible to enhance stress resilience in these symbionts by modulating the expression of protective proteins, potentially mitigating coral bleaching in response to rising ocean temperatures.

## Supplementary Information

Supplementary material 1.

Supplementary material 2.

Supplementary material 3.

## Figures and Tables

**Fig. 6:**
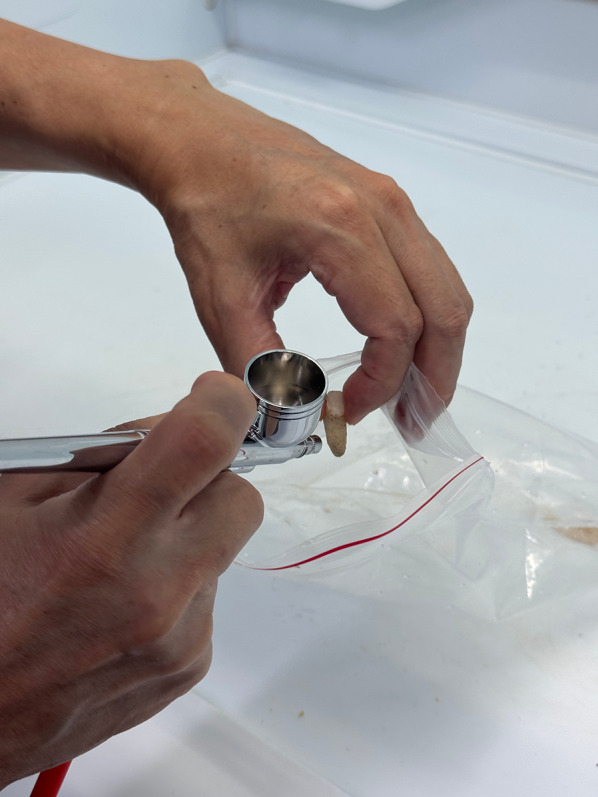
Smashing coral cells to release the symbiotic dinoflagellates using a painter’s airbrush

**Fig. 7:**
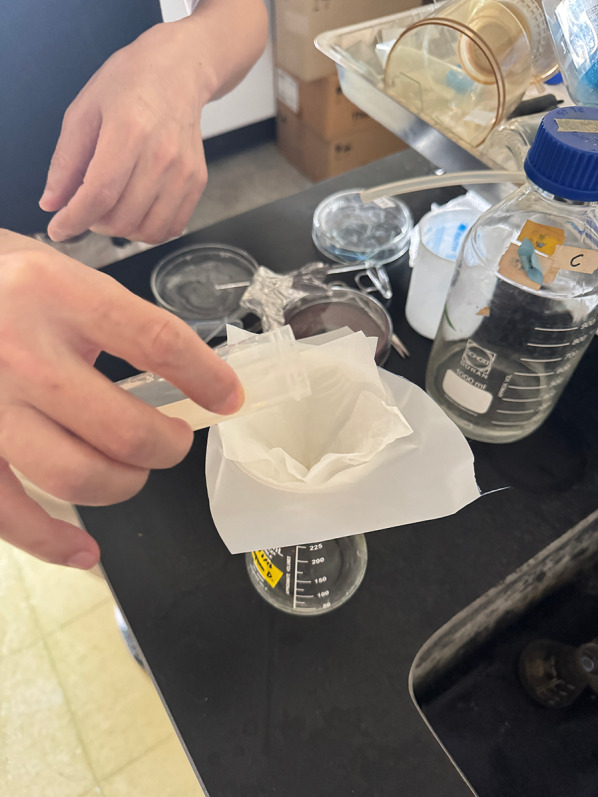
Filtering the mix of coral debris and symbiotic dinoflagellates using a layer of kimwipe paper supported by a layer of nylon filter

## Data Availability

The datasets generated and/or analyzed during the current study are available in the GenBank, under the accession numbers OP881430 and OP881431. The coral symbiotic dinoflagellate clones are available upon request.

## Appendix

See Figs. 6, 7.
