# Supplementary material for: Activation of endogenous tolerance to bleaching stress by high salinity in cloned endosymbiotic dinoflagellates from corals
Source: Bot Stud. 2025 Jan 15;66:3. doi: 10.1186/s40529-025-00451-5 (PMC11735819; doi:10.1186/s40529-025-00451-5)
Supplement: Supplementary file 1 — Supplementary material 1. [file 40529_2025_451_MOESM1_ESM.pdf]

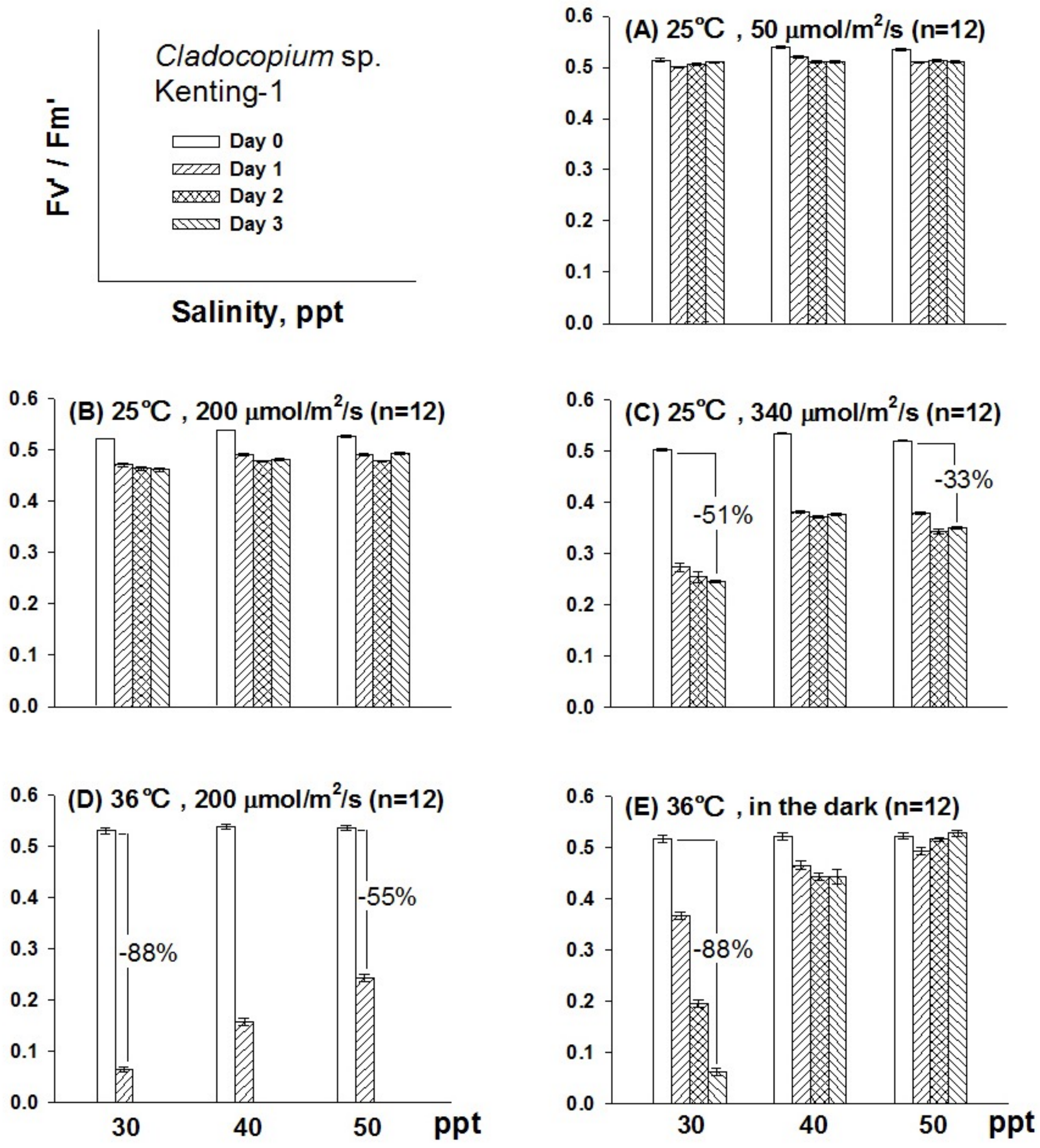

Supplementary Figure 1. Changes in the light-adapted photosystem II (PSII) efficiency (Fv'/Fm') of *Cladocopium* sp. Kenting-1 cells treated under different conditions of temperature, light intensity, and salinity for 3 days (n = 12, mean ± SE). Day/night = 14/10 hrs. (A) – (C), temperature (day/night) at 25 °C; (D) and (E), 36 °C (day) and 28 °C (night). Before the stress treatments, these cells were incubated at 30, 40, or 50 ppt salinity at 25 °C in 50 μmol photon/m^2/s for 3 days.
