# Supplementary material for: Activation of endogenous tolerance to bleaching stress by high salinity in cloned endosymbiotic dinoflagellates from corals
Source: Bot Stud. 2025 Jan 15;66:3. doi: 10.1186/s40529-025-00451-5 (PMC11735819; doi:10.1186/s40529-025-00451-5)
Supplement: Supplementary file 2 — Supplementary material 2. [file 40529_2025_451_MOESM2_ESM.pdf]

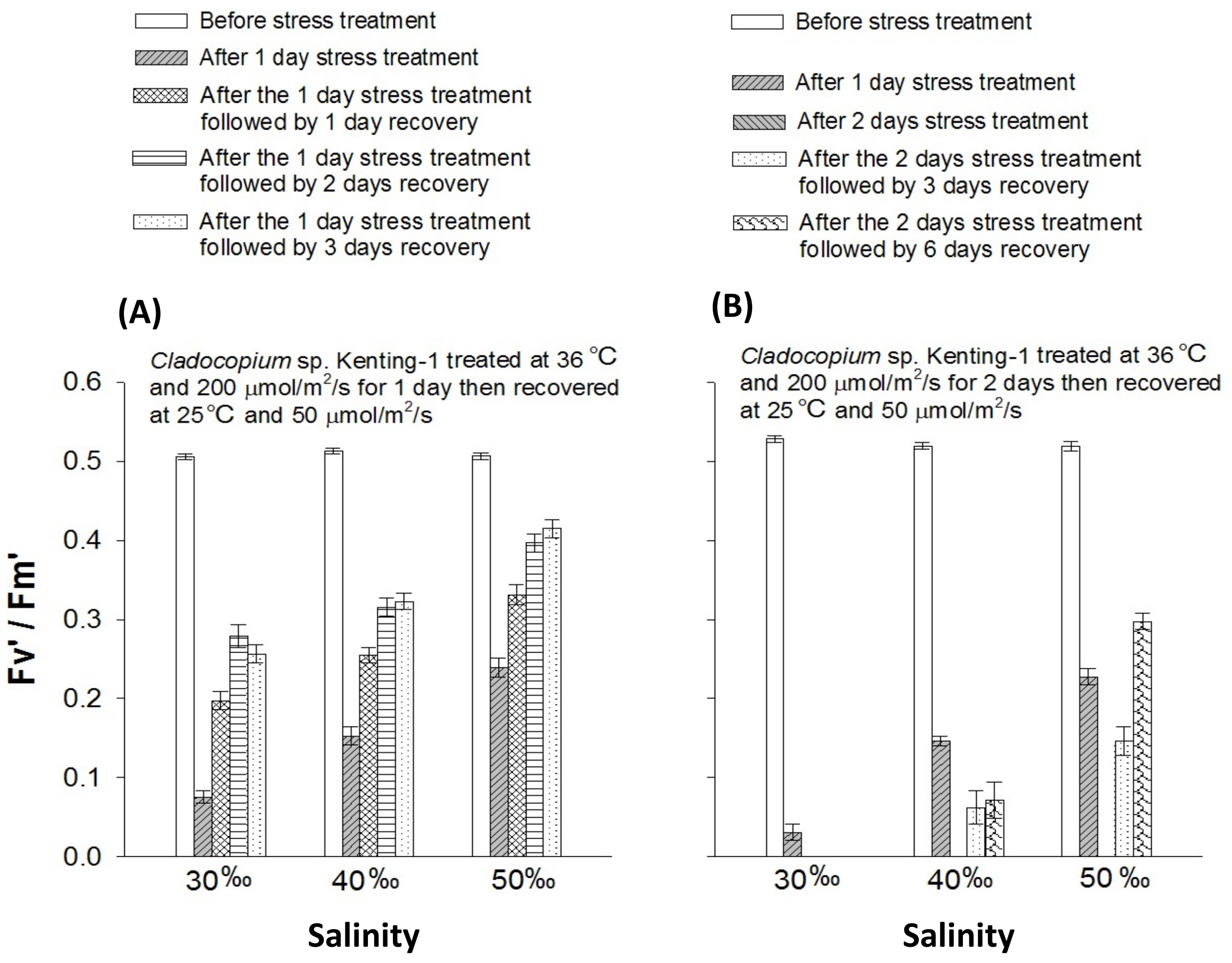

Supplemmentary Figure 2. Recovery of the light-adapted photosystem II (PSII) efficiency (Fv'/Fm') of *Cladocopium* sp. Kenting-1 after the stress treatment (36 °C with 200 μmol photon/m^2/s at daytime and 28 °C at night, day/night = 14/10 hrs) for one day (A) or two days (B) at different levels of salinity (n = 12, mean ± SE).
