# Supplementary material for: Activation of endogenous tolerance to bleaching stress by high salinity in cloned endosymbiotic dinoflagellates from corals
Source: Bot Stud. 2025 Jan 15;66:3. doi: 10.1186/s40529-025-00451-5 (PMC11735819; doi:10.1186/s40529-025-00451-5)
Supplement: Supplementary file 3 — Supplementary material 3. [file 40529_2025_451_MOESM3_ESM.pdf]

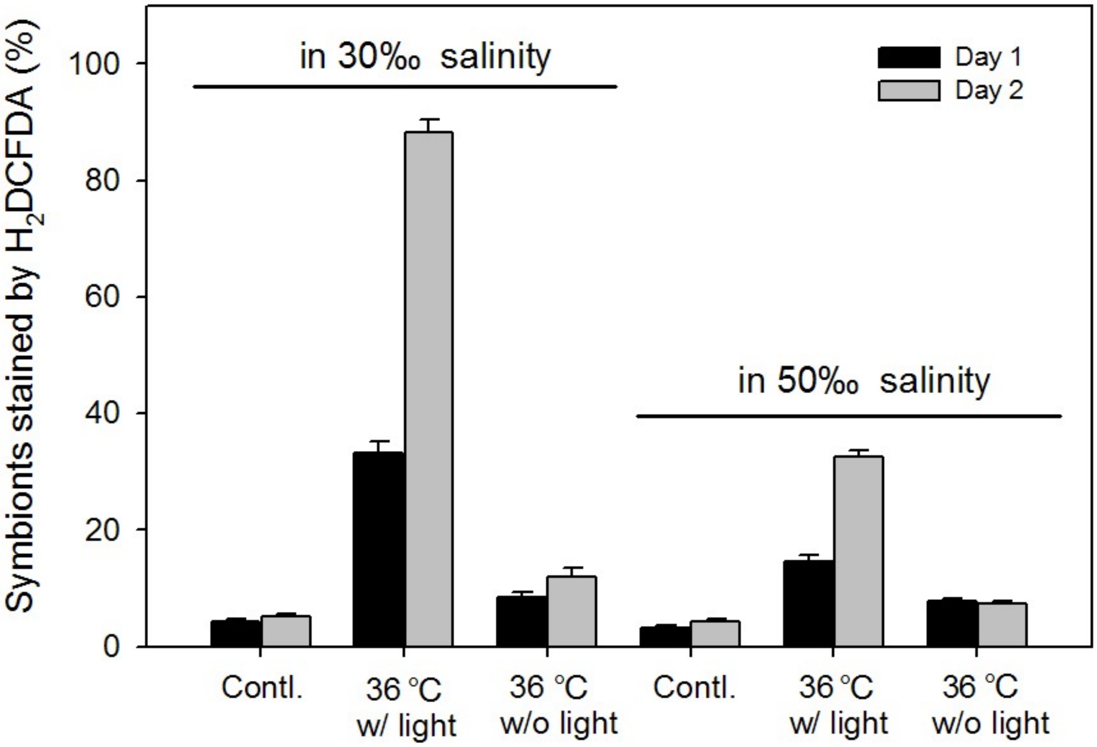

Supplementary Figure 3. Proportions of *Cladocopium* sp. Kenting-1 cells stained by the fluorescent dye H_2DCFDA in different conditions (n = 3, mean ± SE). Cells in the experimental groups were treated at 30 or 50 ppt salinity coupled with (w/) 200 μmol photon/m^2/s light intensity or without (w/o) light at 36 °C. The control groups (Contl.) were treated at 25 °C coupled with 50 μmol photon/m^2/s light intensity at 30 or 50 ppt salinity.
